# Druitt’s Surgeon’s Vade-Mecum

**Published:** 1887-12

**Authors:** 


### Book Reviews.

---

Druitt's Surgeon's Vade-mecum. Edited by Stanley Boyd, M. B., London, Assistant Surgeon Charing Cross Hospital, etc. Twelfth Edition. Philadelphia: Lea Brothers & Co. 1887.

In the editor's preface mention is made of the great progress of surgery since the appearance of the last edition of this popular work. Consequently Mr. Boyd is justified in leaving scarcely a paragraph of the eleventh edition unaltered. Such wholesale alterations were very necessary in order to make the book what it purports to be, viz., "A Manual of Modern Surgery," but it seems to us that the editor should have gone a step further and altered the name of the book as well, for it is no longer Druitt's. When Mr. Boyd entered on his task of re-writing this work, he should have made an effort to bring the *whole* book up to the high standard of excellence of certain parts of it. In other words, some chapters (many, indeed) are fully abreast of the times, while others are woefully behind. The opening chapter, "The Etiology of Disease," contains an excellent *résumé* of our present knowledge on the subject of microbes in their relation to disease, the action of antiseptics and their relative germicidal power. Tuberculosis receives thoughtful consideration, the editor accepting all of the results which Koch reached and fully committing himself to the opinion that the "bacillus tuberculosis is the specific irritant productive of tubercle."

Tumors are described in twenty pages, and although their description forms a most readable chapter, it seems to us that, considering their great practical importance, more space should have been devoted to them.

The author gives his adherence to the antiseptic method in the treatment of wounds, but he gives a very meagre and unsatisfactory account of the application of this method; it is true he enters into all the details of Listerian dressings, but these have long since become obsolete.

The subject of fractures is, on the whole, very well discussed, but Mr. Boyd seems to be entirely ignorant of a number of improvements in the treatment of special fractures originated by American surgeons; for instance, the interdental splint in fractures of the lower jaw, and Buck's extension apparatus in fractures of the femur. He does speak of the use of Dr. Buck's apparatus in fractures of the leg, a use to which it is rarely, if ever, put in this country.

Colles' fracture is dismissed with a much shorter notice than its frequency and importance demand.

On the subject of stricture of the urethra, Mr. Boyd overlooks entirely the very numerous and valuable additions which American surgeons have made to our knowledge of this disease.

The chapter on abdominal surgery is one of the best in the book, and is fully in accord with the progressive spirit of the present day. A succinct account of such modern operations as nephrotomy, nephrolithotomy, nephrectomy and nephrorraphy will be found in the chapter on the surgery of the kidney. The description of the ligature of the differenf arteries is especially to be commended.

All of the defects of the work have been mentioned; excluding these it is as good as any text-book could well be.

